# Microbiological, Epidemiological, and Clinical Characteristics of Patients With Cryptococcal Meningitis at a Tertiary Hospital in China: A 6-Year Retrospective Analysis

**DOI:** 10.3389/fmicb.2020.01837

**Published:** 2020-07-29

**Authors:** Yanbing Li, Mingxiang Zou, Jun Yin, Ziqing Liu, Binghuai Lu

**Affiliations:** ^1^Department of Laboratory Medicine, Xiangya Hospital, Central South University, Changsha, China; ^2^Department of Neurology, Xiangya Hospital, Central South University, Changsha, China; ^3^Laboratory of Clinical Microbiology and Infectious Diseases, Department of Pulmonary and Critical Care Medicine, National Clinical Research Center for Respiratory Diseases, China-Japan Friendship Hospital, Beijing, China; ^4^Guangdong Key Laboratory for Emerging Infectious Diseases, National Clinical Research Center for Infectious Diseases, Shenzhen Third People’s Hospital, Southern University of Science and Technology, Shenzhen, China

**Keywords:** cryptococcal meningitis, *C. neoformans* species complexes, *C. gattii* species complexes, antifungal susceptibility testing, cryptococcosis

## Abstract

Cryptococcal meningitis, mainly caused by *Cryptococcus neoformans/gattii* species complexes, is a lethal infection in both immunosuppressive and immunocompetent populations. We characterized 110 *Cryptococcus* strains collected from Xiangya Hospital of Central South University in China during the 6-year study period between 2013 and 2018, and performed their antifungal susceptibility testing. Furthermore, the clinical features, laboratory and imaging data, treatment strategies and outcomes of the subjects were retrospectively analyzed. Of 110 *Cryptococcus* strains, *C. neoformans* species complexes accounted for 96.4% (106/110), including *C. neoformans sensu stricto* (VNI molecular type, 95.5%, 105/110) and *Cryptococcus deneoformans* (VNIV molecular type, 0.9%, 1/110), and *Cryptococcus deuterogattii* (VGII molecular type) accounted for 3.6% (4/110). The strains were further classified into 17 individual sequence types (STs) by using multilocus sequence typing (MLST). 89.1% (98/110) were represented by ST5; seven *C. deuterogattii* strains and one *Cryptococcus deneoformans* strain were assigned as ST7 and ST260, respectively. Antifungal minimal inhibitory concentrations above the epidemiological cutoff values (ECVs) were found mainly in *C. neoformans* species complexes strains (nine for amphotericin B, nine for fluconazole and seven for 5-fluorocytosine). Furthermore, 60.9% (67/110) of the subjects were male, and 40.0% (44/110) did not have underlying diseases. Hepatic diseases (hepatitis/HBV carrier status and cirrhosis) were the most common underlying health conditions (11.8%, 13/110), followed by autoimmune disorders (10.9%, 12/110) and chronic kidney disease (6.36%, 7/110). Only 4.5% (5/110) of the patients were HIV/AIDS positives. For clinical presentation, headache (77.3%, 85/110), fever (47.3%, 52/110), and stiff neck (40.9%, 45/110) were commonly observed. The mortality rate was 35.0% (36/103). In conclusion, our data were characterized by a high prevalence of the Cryptococcal meningitis patients without HIV/AIDS and other underlying health conditions, a relatively high non-wild-type rate of fluconazole and amphotericin B resistance, and low genetic diversity in *Cryptococcus* strains. The present study will provide evidence for further improvement of the diagnosis and treatment of cryptococcosis in China.

## Introduction

Cryptococcosis is an opportunistic and potentially life-threatening infection, not only occurring in immunocompromised patients, including those with HIV/AIDS and autoimmune diseases, and transplant recipients, but also posing threat to apparently immunocompetent subjects (Bratton et al., 2012; Kwon-Chung et al., 2017; Beardsley et al., 2019; Ellis et al., 2019). The current genus *Cryptococcus* contains 10 species, of which seven are pathogenic to humans and animals: the two members of the *Cryptococcus neoformans* species complex that are *C. neoformans sensu stricto* (serotype A; AFLP1/VNI, AFLP1A/VNB/VNII, AFLP1B/VNII), *Cryptococcus deneoformans* (serotype D; AFLP2/VNIV); and the six species in the *Cryptococcus gattii* species complex: *Cryptococcus gattii sensu stricto* (serotype B; AFLP4/VGI), *Cryptococcus bacillisporus* (serotype B&C; AFLP5/VGIII), *Cryptococcus deuterogattii* (serotype B; AFLP6/VGII), *Cryptococcus tetragattii* (serotype C; AFLP7/VGIV) and *Cryptococcus decagattii* (AFLP10/VGIV/VGIIIc) (Hagen et al., 2015, 2017; Kwon-Chung et al., 2017; Ashton et al., 2019). Furthermore, a new lineage of *Cryptococcus gattii* species complex (serotype B, VGV) was discovered by Farrer et al. in 2019, while cryptococcosis by other species has rarely been documented till date (Farrer et al., 2019). Multilocus sequence typing (MLST) based on seven housekeeping genes allows for the classification of most clinical *Cryptococcus* strains into varied sequence types (STs), among which ST5 is mainly reported from mainland China (Fan et al., 2016).

The genus *Cryptococcus* usually invades the central nervous system (CNS) and results in cryptococcal meningitis and then a high mortality rate (Lahiri et al., 2019). In addition, meningitis by *C. neoformans* species complex occurred more frequently than that by *C. gattii* species complex (Yuchong et al., 2012; Hagen et al., 2015; Smith et al., 2015; Ferreira-Paim et al., 2017; Thanh et al., 2018; Rakotoarivelo et al., 2020), and this might be explained by that the former is globally distributed, while the latter seems to be geographically restricted (Kidd et al., 2007; Chen et al., 2014; Smith et al., 2015; Firacative et al., 2016; May et al., 2016; Souto et al., 2016). Furthermore, both amphotericin B and fluconazole remain the mainstay treatment in cryptococcal meningitis (Yao et al., 2014; Beardsley et al., 2019). The resistance patterns to them are documented worldwide, and geographic variability is noted (Fan et al., 2016; Nyazika et al., 2016).

Understanding the epidemiological characteristics of local *Cryptococcus* strains and clinical features of cryptococcal meningitis is essential for the development of efficient diagnosis and treatment strategy. Studies on that are rare in China (Fan et al., 2016; Guo et al., 2016; Liu et al., 2017; Cao et al., 2019), and they focused on pediatric patients (Guo et al., 2016), clinical features (Liu et al., 2017; Cao et al., 2019), or molecular and antifungal resistance characteristics (Fan et al., 2016), respectively. Continuous and comprehensive monitoring of the epidemical changes is crucial for the treatment and prevention of cryptococcosis. The present study involved 110 cryptococcal meningitis cases from 2013 to 2018 in Xiangya Hospital of Central South University (XHCSU), Hunan, China. The molecular characteristics and antifungal agent susceptibility data of *Cryptococcus* strains, and the clinical, demographic features and therapeutic outcomes were documented to help improve timely diagnosis and reduce the mortality rate.

## Materials and Methods

### Ethical Approval

The institutional review boards at the XHCSU approved the study protocol. The written informed consent from participants was waived and the data were analyzed anonymously.

### Biosafety Procedures

The procedures, including the incubation, nucleic acid extraction, susceptibility testing of *Cryptococcus* strains, were performed in a Class II, Type B2 biological safety cabinet (LB2-5B1, ESCO, Singapore) in a biosafety level 2 laboratory. Furthermore, additional biosafety precautions, including masks and gloves, were taken.

### Case Definition

A case of culture-confirmed cryptococcal meningitis was defined as the isolation of *Cryptococcus* strains from cerebrospinal fluid (CSF). Immunocompromised status was defined as the following: HIV/AIDS, transplant recipient, diabetes mellitus, malignancy, glucocorticosteroid treatment, hepatic diseases (hepatitis B virus-carrier, cirrhosis, and chronic liver failure), etc.

### Demographic and Clinical Features of Cryptococcal Meningitis Cases

*Cryptococcus* strains were obtained from CSF in 110 patients at XHCSU, China, between January 2013 and December 2018.

The clinical manifestations, laboratory variables and demographic characteristics of these above patients were retrospectively reviewed, including the following variables: demographic characteristics (age, sex, and place of residence), symptoms (headaches, altered mental status, fever, speech difficulties, and others), and laboratory and imaging examination results, underlying diseases, suspected exposure to pigeon excrements, main antifungal regimen, laboratory results, and surgical treatment regimen (use of ventriculoperitoneal shunt). All 110 patients, except for seven lost to follow-up cases, were categorized as survival or death during a 1-year follow-up.

### Strains Collection and Primary Identification by Using Matrix Assisted Laser Desorption Ionization-Time of Flight Mass Spectrometry (MALDI-TOF MS)

All *Cryptococcus* strains were streaked onto Sabouraud medium, and incubated at 35°C for 24 h or more if necessary. The fresh colonies were collected and identified on the basis of colony morphology and MALDI-TOF MS (Bruker Daltonik, Bremen, Germany) according to the manufacturer’s suggested recommendations. The identification was matched with the Bruker spectra library program (version 4.0.0.1, 5,627 entries), preinstalled in the Bruker Biotyper device (version 3.1; Bruker.1). Manufacturer-recommended identification score criteria were used: a score of ≧2.000 indicated an identification to species-level, a score of 1.700 to 1.999 indicated to the genus level, and a score of <1.700 was interpreted as no identification.

The isolates were identified firstly by using a direct transfer method. Briefly, fresh colonies were picked up with an inoculation loop, smeared on an MTP 384 steel target plate, coated with a matrix solution of α-cyano-4-hydroxycinnamic acid (HCCA) in 50% acetonitrile with 2.5% trifluoroacetic acid (TFA), and dried at room temperature. If no reliable result was obtained, an ethanol/formic acid extraction method was then applied. One loop of fungal mass was suspended in de-ionized water (300 μl), and pure ethanol (900 μl) was added. The suspension was mixed for 1 min using a vortex mixer. The cell suspension was centrifuged (13,000 rpm for 2 min). The supernatant was discarded. Then, the pellet was dried and resuspended with 70% formic acid (50 μl) with thorough mixing, and then 50-μl acetonitrile was added. After centrifugation (13,000 rpm for 2 min), the 1-μl pellet was applied on a steel target plate, dried at room temperature, and coated with HCCA (1 μl).

### DNA Extraction

Genomic DNA was extracted from each *Cryptococcus* strain following the protocol described by Chen et al. (2018), with some modifications. Protoplasts were prepared by incubating the above-mentioned fresh *Cryptococcus* strains in a microcentrifuge tube with 1-ml saline, and the solution was prepared to a concentration of 2 McFarland, and centrifuged at 12,000 rpm for 1 min. The supernatant was discarded. We added 600-μl PBS buffer and 6-μl (10 U/μl) cell wall breaking enzyme (Tiangen biochemical technology co., Ltd., China) into microcentrifuge tube, and thoroughly mixed and incubated it at 37°C for 120 min. After vortexing, 400 μl of 2-μm acid-washed glass beads were added and further vortexed. Extracted DNAs were dissolved in TE buffer and stored at -20°C until used as PCR templates.

### Internal Transcribed Spacer (ITS) Sequencing

Identification of *Cryptococcus* species through the amplification of the specific ITS region was performed using two universal primers ITS1 and ITS4 (ITS1: 5′-TCCGTAGGTGAACCTGCGG-3′ and ITS4: 5′-TCCTCCGCTTATTGATATGC-3′), as described previously (Nascimento et al., 2016). The PCR products were sequenced in both directions and were compared against those contained in the Centraalbureau voor Schimmelcultures (CBS) hosted at the Westerdijk Fungal Biodiversity Institute^1^. Furthermore, the sequences were aligned in line with reference sequences of *Cryptococcus* type strain of H99 (*C. neoformans s.s.*, VNI, GenBank accession number KY102799), CBS 8710 (*C. neoformans s.s.*, VNI, NR130682), WM 148 (*C. neoformans s.s.*, VNI, KY102824), WM 626 (*C. neoformans s.s.*, VNII, KY102823), WM 628 (*C. neoformans s.s.*, VNIII, FJ914893), JEC20 (*C. deneoformans*, VNIV, KY102637), JEC21 (*C. deneoformans* serotype D, VNIV, AE017342), CBS 8273 (*C. gattii*, VGI, NR144805), WM 178 (*C. deuterogattii*, VGII, KY102659), WM 161 (*C. bacillisporus*, VGIII, KY102615), CBS 11249 (*C. tetragattii*, VGIV, KY102969), downloaded from the GenBank database to infer species boundaries and identify the *Cryptococcus* strains to species level.

### Multi-Locus Sequence Typing (MLST)

MLST was performed to identify the molecular type of *Cryptococcus* strains using the consensus scheme established by the Cryptococcal Working Group of the International Society for Human and Animal Mycology (ISHAM) *via* amplifying and sequencing the internal fragments within seven housekeeping gene loci (namely, *CAP59*, *GPD1*, IGS1, *LAC1*, *PLB1*, *SOD1*, and *URA5*), as described previously (Meyer et al., 2009). The PCR products were sequenced in both directions. The allelic numbers and sequence types (STs) were further identified by querying the online MLST database^2^. Molecular types (i.e., VNI to VNIV for *C. neoformans* species complex and VGI to VGIV for *C. gattii* species complex) were assigned according to their allelic numbers and STs. The ST was used to infer phylogeny. Briefly, jModelTest software was used to select the algorithm that best fit our data. Of 88 models, TIM1 + G algorithm demonstrated the lowest Akaike information criteria (AIC) value (Guindon and Gascuel, 2003; Darriba et al., 2012). Then, the phylogeny tree was constructed with IQ-TREE software and iTOL v4^3^ by using the TIM1 + G model (Nguyen et al., 2015; Kumar et al., 2018). The bootstrap value was set to 1,000.

### Antifungal Susceptibility Testing

The micro-broth dilution method (Sensititre YeastOne colorimetric plate, Thermo Fisher Scientific, MA, United States) was used to determine the susceptibility of all *Cryptococcus* strains to the six antifungal drugs, namely, fluconazole, 5-fluorocytosine, amphotericin B, itraconazole, posaconazole, and voriconazole. The procedures followed the manufacturer’s instructions. Two well-trained microbiologists read plates and interpreted the endpoints for the antifungals. The results were reported as wild-type (WT) or non-wild-type (non-WT) in accordance with the epidemiological cutoff value (ECV) set for *Cryptococcus* spp. by the Clinical and Laboratory Standards Institute (Espinel-Ingroff et al., 2012a, b; CLSI, 2018). Given no recommended ECV for *C. deneoformans*, except for voriconazole (ECV = 0.12 μg/ml) recommended by Espinel-Ingroff et al., the minimum inhibitory concentration (MIC) value was used for the following analysis (Espinel-Ingroff et al., 2012a).

### Statistical Analysis

We evaluated the differences among groups *via* the Mann-Whitney U test or *t*-test for continuous variables (expressed as the median [IQR]) or mean value ± standard deviation (Std.) and χ2 tests for categorical variables, as appropriate. Statistical analyses were conducted using GraphPad Prism version 8.0.1. A *p* value of less than 0.05 was considered statistically significant. MIC data were recorded and analyzed by WHONET 5.6 software, and MIC50 and MIC90 were also calculated.

## Results

### Demographic Features of 110 Cryptococcal Meningitis Patients

**FIGURE 1:**
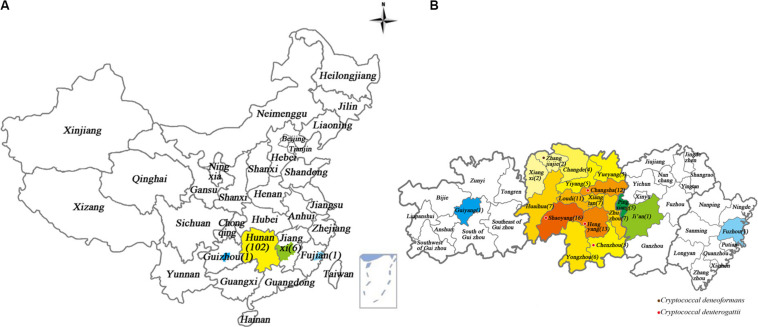
Geographical locations of *Cryptococcus* strains from patients diagnosed with cryptococcal meningitis in Xiangya Hospital, Hunan, China, between 2013 and 2018. The color-highlighted provinces, cities and counties represent those where *Cryptococcus* strains were recovered, with the number of strains shown in brackets. **(A)** China; **(B)** Provinces where patients came from, including Hunan, Jiangxi, Fujian, and Guizhou provinces. Purple dot: where one *C. deneoformans* strain was detected. Red dot: where four *C. deuterogattii* strains were detected.

**FIGURE 2:**
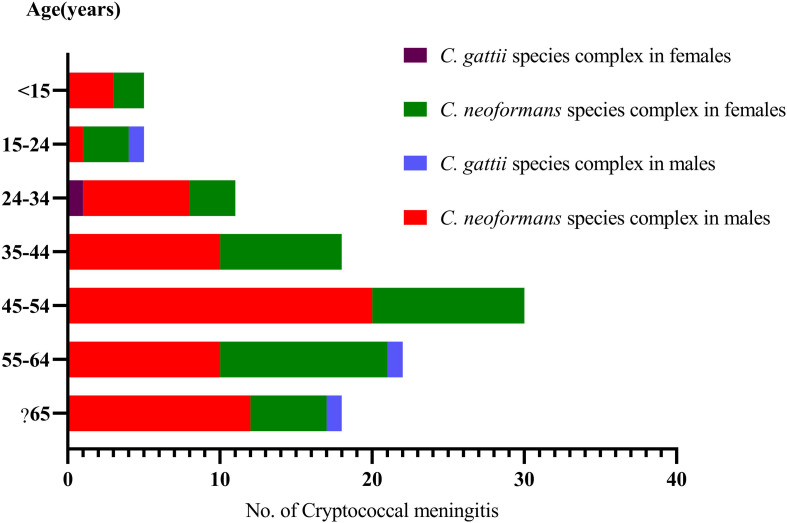
Distribution of the sex and ages in 110 cryptococcal meningitis cases in a tertiary hospital in China.

**FIGURE 3:**
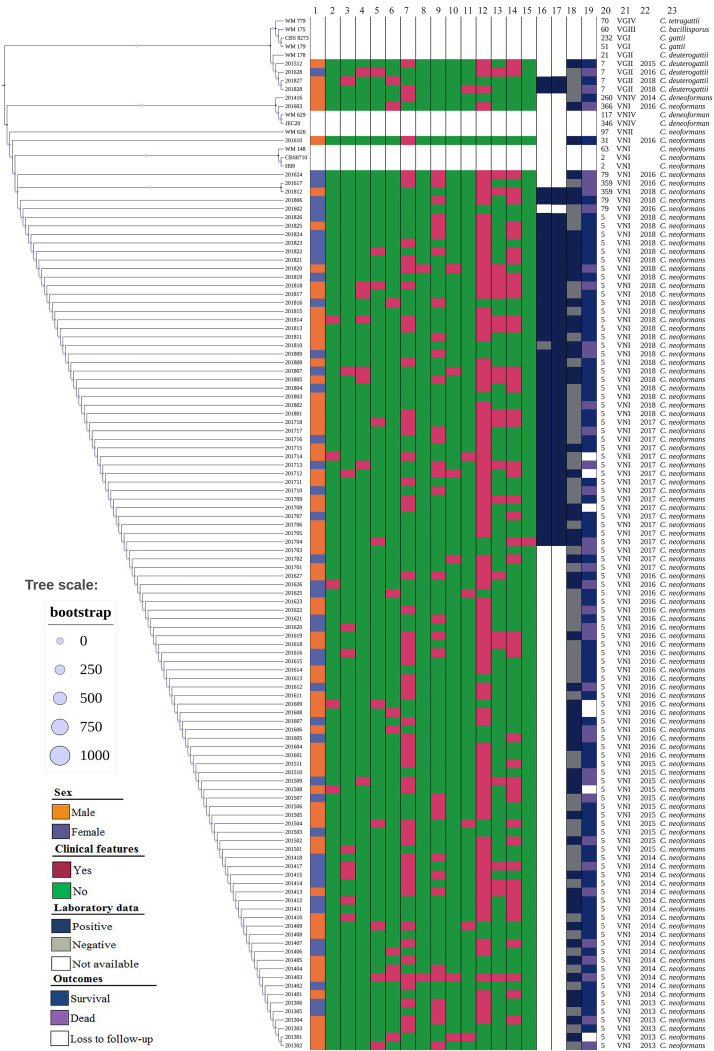
The phylogenetic tree diagram of 110 clinical unique strains of *Cryptococcus* and various type strains based on MLST sequences: *C. neoformans s.s.* (105 strains) and *C. deneoformans* (1 strain), and *C. deuterogattii* (4 strains) clustered into different groups. Reference sequences of VNI, VNII, VNIII, VNIV, and VGII as outgroups are included. From left to right: 1 Sex; 2–15, clinical features: 2 Speech difficulties; 3 Visual disturbance; 4 Brinell sign; 5 Altered mental status; 6 Dizziness; 7 Fever/chill; 8 Hemiplegia; 9 Nausea/vomiting; 10 Palsies; 11 Unstable walking; 12 Headache; 13 Klinefelter sign; 14 Nick stiffness; 15 Sepsis shock; 16∼18 Laboratory data:16 CSF cryptococcal antigen; 17 Blood cryptococcal antigen; 18 India ink staining; 19 Outcomes; 20 Sequence type (ST); 21 Molecular type; 22 Year of isolation of Cryptococcus strains; 23 Cryptococcus species.

The demographic features of the subjects were shown in Table 1 and Figures 1–3.

**TABLE 1:** Epidemiological characteristics of 110 patients with cryptococcal meningitis.

Demographic features	No.	%	*C. neoformans*	*C. gattii*	*p*
Total	110		106	4	
**Gender**					1.000
Male	67	60.9	64	3	
Female	43	39.1	42	1	
**Ages (years)**					0.230
≤14	5	4.6	5	0	
15–24	5	4.6	4	1	
25–34	12	10.9	11	1	
35–44	18	16.4	18	0	
45–54	30	27.3	30	0	
55–64	22	20.0	21	1	
≧65	18	16.4	17	1	
**Duration from the onset of symptoms to diagnosis**					0.624
<2 w	22	20.0	22	0	
2 w–6 m	85	77.2	81	4	
>6 m	3	2.7	3	0	
**Underlying status**					1.0
No underlying conditions	44	40.0	40	4	
Hepatitis and liver cirrhosis	13	11.8	13	0	
Autoimmune disorders	12	10.9	12	0	
CKD	7	6.4	7	0	
HIV/AIDS	5	4.6	5	0	
Diabetes	5	4.6	5	0	
Immunosuppressants	3	2.7	3	0	
Malignancy	2	1.8	2	0	
Pregnancy	2	1.8	2	0	
Transplant recipient	1	0.9	1	0	
Other underlying conditions*	16	14.6	16	0	
**Contact with pigeon droppings**	9	8.2	9	0	0.296

*Includes hypertension, coronary heart disease, tuberculosis, chronic obstructive pulmonary disease; CKD, Chronic kidney disease.

The 110 subjects were from Hunan (102, 92.7%), Jiangxi (6 cases, 5.5%), Fujian (1, 0.9%), and Guizhou (1, 0.9%) in China. The detailed residence places and numbers of the cryptococcal meningitis cases were illustrated in Figure 1.

On average, we received 18 strains each year (2013, 6; 2014, 18; 2015, 12; 2016, 28; 2017, 18; 2018, 28). Our patients aged 47.7 ± 16.5 years, ranging from 7 to 78 years, 60.9% (67/110) were males, and 16.4% (18/110) were classified as being elderly (≧65 years, 13 males and 5 females). Furthermore, in 106 cases with *C. neoformans* species complex infection, 60.4% (64/106) were male. Four *C. gattii* species complex strains were isolated from three male patients (75%), aged 20, 58 and 66 years, respectively, and one female (25%), aged 28 years.

### Distribution of *Cryptococcus* Species by ITS Sequencing

Of 110 *Cryptococcus* strains, in line with ITS sequence and MLST results, *C. neoformans* species complex was the predominant species (96.4%, 106/110, including 105 *C. neoformans s.s.* (VNI molecular type) and one *C. deneoformans* (VNIV molecular type), and four *C. deuterogattii* (VGII molecular type) were rarely identified (3.6%). This is illustrated in Figure 3 and Supplementary Figure S1. The ITS sequences and seven MLST loci-sequences of these 110 *Cryptococcus* strains are deposited at GenBank under the accession no. MT437078 to MT437187, and MT474939 to MT475708, respectively.

### Low Genetic Diversity as Shown by MLST Analysis

Only seven sequence types (STs) were distinguished among 110 *Cryptococcus* strains. As illustrated in Figure 3, four *C. deuterogattii* strains and one *C. deneoformans* strain were assigned as ST7 and ST260, respectively. Moreover, 89.1% were represented by ST5 that has been shown to be widely distributed in mainland China, and other four STs were ST79 (3, 2.7%), ST359 (2, 1.8%), ST31 (1, 0.9%), and ST366 (1, 0.9%).

### Clinical Presentation

Of 110 cases, 60.0% had underlying conditions, in which hepatitis and cirrhosis were the most common (13, 11.8%), followed by autoimmune disorders (12, 10.9%), chronic kidney disease (CKD, 7, 6.4%), HIV/AIDS (5, 4.5%), and diabetes mellitus (5, 4.5%). No patients with underlying status were infected by *C. gattii* species complex strains. Nine cases (9/110, 8.2%) have pigeon droppings contact history. These were shown in Table 1.

With regards to the clinical presentations, the headache was the most common (85, 77.3%), followed by fever (52, 47.3%), stiff neck (45, 40.9%), and nausea/vomiting (32, 29.1%), as shown in Table 2 and Figure 3.

**TABLE 2:** Clinical presentations of 110 cryptococcal meningitis subjects with or without underlying diseases.

Presentations	Total (*n* = 110)	Percent	Subjects with underlying diseases (*n* = 66), No. %		Previously healthy subjects (*n* = 44), No. %		*p*
Headache	85	77.3	49	72.2	36	81.8	0.353
Fever/chill	52	47.3	32	48.5	20	45.5	0.755
Stiff neck	45	40.9	22	33.3	23	52.3	0.048^#^
Nausea/vomiting	32	29.1	16	24.2	16	36.4	0.17
Klinefelter	23	20.9	10	15.2	13	29.6	0.069
Dizziness	10	9.1	5	7.6	5	11.4	0.498
Altered mental status	10	9.1	6	9.1	4	9.1	1
Visual disturbance	10	9.1	3	4.6	7	15.9	0.042^#^
Brinell sign	8	7.3	3	4.6	5	11.4	0.177
Palsies	6	5.5	3	4.6	3	6.8	0.607
Speech difficulties	5	4.6	3	4.6	2	4.6	1
Unstable walking	5	4.6	2	3.0	3	6.8	0.35
Hemiplegia	2	1.8	2	3.0	0	0	0.244
Seizures	1	0.9	0	0	1	2.3	0.412
Septic shock	1	0.9	1	1.5	0	0	0.412

**^#^p < 0.05.**

In line with the data reviewed, in 43 culture-confirmed cryptococcal meningitis cases in whom cryptococcal antigen tests have been conducted in both CSF and blood samples, 43 (100%) and 42 (97.7%) were blood and CSF positive, respectively. However, India ink staining of CSF has been conducted in all 110 cases, and only 53 (48.2%) CSF specimens were positive.

### Chest CT and Cranial Magnetic Resonance Imaging (MRI) Examination

The chest CT examination revealed that there were no obvious abnormalities in 37 cases (33.6%). Ground-glass opacity and patchy shadow were the most common pulmonary lesions (33 cases, 30.0%). Single-shot nodules in the right lobe (14 cases, 12.7%) were mostly commonly in those with nodules, followed by double lung nodule (12, 10.9%) and left lung nodule (5, 4.6%). Stripe-like opacities were found only in 6 (5.5%) cases. The coexistence of the nodule and cavity was detected in three (2.7%) cases.

Furthermore, of 110 cases, abnormal changes in head MRI were observed in 91 cases (82.7%). The mostly observed abnormalities were multi-site lesions. The details are shown in Table 3.

**TABLE 3:** Characteristics of magnetic resonance imaging (MRI) examination results in 110 patients with cryptococcal meningitis.

MRI characteristics	No.	%
Local lesions		
Frontal lobe	15	13.6
Brain stem	4	3.6
Basal ganglion	5	4.6
Cerebral ventricle	2	1.8
Occipital region	1	0.9
Multi-site lesions	33	30.0
Abnormal meningeal enhancement	2	1.8
Hydrocephalus	7	6.4
Focal lesions + hydrocephalus	6	5.5
Focal lesions + meningeal enhancement	6	5.5
Hydrocephalus + meningeal enhancement	1	0.9
Focal lesions + ventricular dilatation	4	3.6
Focal lesions + ventricular dilatation + meningeal enhancement	4	3.6
Ventricular dilatation + hydrocephalus + meningeal enhancement	1	0.9
No lesions	19	17.3

### Antifungal Susceptibility Results

**FIGURE 4:**
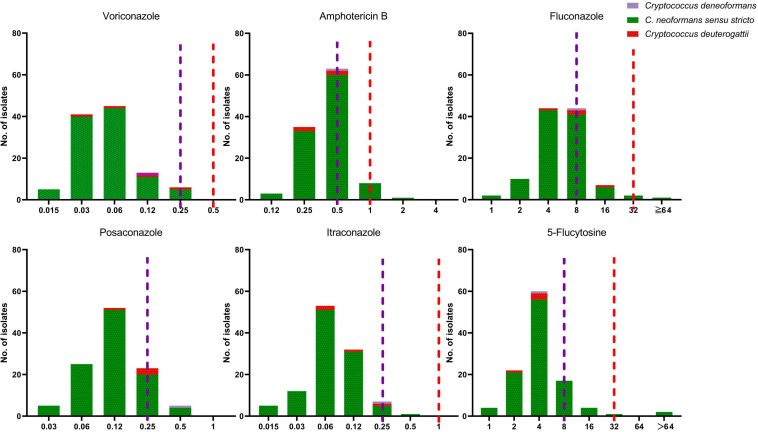
The distribution of the minimum inhibitory concentration (MIC). 110 clinical unique strains of *C. neoformans s.s.* (VNI molecular type) (105 strains), *C. deneoformans* (VNIV) (1 strain), *C. deuterogattii* (VGII) (4 strains) for six antifungal agents. Purple line: the epidemiological cutoff value (ECV) for *C. neoformans s.s.* (VNI molecular type); Red line: ECV for *C. deuterogattii* (VGII).

The antifungal susceptibility and MIC results for six agents tested against all 110 *Cryptococcus* strains, including MIC50, MIC90 and MIC range, are presented in Table 4 and Figure 4. Our results demonstrate that all four *C. deuterogattii* strains were uniformly WT to the studied antifungal agents. In contrast, 8.6% (9/105), 6.6% (7/105), 8.6% (9/105), 3.8% (4/105), and 1.0% (1/105) of *C. neoformans s.s.* strains were non-WT to fluconazole, 5-fluorocytosine, amphotericin B, posaconazole, and itraconazole, respectively, but all strains were WT to voriconazole. The MICs of *C. deneoformans* (VNIV) strain (201416) for fluconazole, 5-fluorocytosine, voriconazole and amphotericin B were 8, 4, 0.12, and 0.5 μg/ml, respectively. There is no ECV for *C. deneoformans* against antifungal agents recommended by CLSI-M59 (CLSI, 2018), however, in line with the study results on ECV of *C. neoformans/gattii* species complex by Espinel-Ingroff et al., the ECV ranges were recommended as follows, fluconazole (8–32 μg/ml), 5-flucytosine (4–16 μg/ml), and amphotericin B (0.5–1 μg/ml), and the EVC for voriconazole was 0.12 μg/ml (Espinel-Ingroff et al., 2012a, b). Therefore, the *C. deneoformans* (201416) strain in the present study was designated as WT to fluconazole, 5-fluorocytosine, voriconazole, and amphotericin B.

**TABLE 4:** Antifungal susceptibility and MIC distribution of 110 *Cryptococcus* strains in a tertiary hospital in mainland China.

	VNI (105)						VGII (4)						VNIV (1)
	ECV	WT	Non-WT	MIC50 (mg/L)	MIC90 (mg/L)	Range (mg/L)	ECV	WT	Non-WT	MIC50 (mg/L)	MIC90 (mg/L)	Range (mg/L)	MIC (mg/L)
Fluconazole	8	96 (91.4%)	9 (8.6%)	4	8	1.0–64.0	32	4 (100%)	0	8	16	4.0–16.0	8
5-Fluorocytosine	8	98 (93.3%)	7 (6.6%)	4	8	1.0–64.0	32	4 (100%)	0	4	4	2.0–4.0	4
Amphotericin B	0.5	96 (91.4%)	9 (8.6%)	0.5	0.5	0.12–2.0	1	4 (100%)	0	0.25	0.5	0.25–0.5	0.5
Posaconazole	0.25	101 (96.2%)	4 (3.8%)	0.12	0.25	0.03–0.5	NA	NA	NA	0.12	0.25	0.12–0.25	0.5
Itraconazole	0.25	105 (99.0%)	1 (1.0%)	0.06	0.12	0.015–0.5	1	4 (100%)	0	0.06	0.25	0.06–0.25	0.25
Voriconazole	0.25	105 (100%)	0	0.06	0.12	0.015–0.25	0.5	4 (100%)	0	0.06	0.25	0.03–0.25	0.12

**MIC, minimum inhibitory concentration; WT, wild-type; Non-WT, non-wild-type; ECV, the epidemiologic cutoff value; NA, not available; MIC50, the minimum inhibitory concentration at which 50% of isolates were inhibited; MIC90, the minimum inhibitory concentration at which 90% of isolates were inhibited; MIC range, range of minimum inhibitory concentration.**

### Treatment and Outcomes

During the study period, preferred induction antifungal therapy in 110 cryptococcal meningitis patients is an amphotericin B plus 5-flucytosine for 2–6 weeks depending on patients’ conditions, followed by consolidation/maintenance therapy with fluconazole for 12 months or longer. Of them, the median duration of disease was 36.6 days. There are three cases who were hospitalized after over 6-month recurrent headaches, and the other107 patients admitted within 6 months after the onset of symptoms. Moreover, 12.7% (14/110) died within 3 days of admission before the establishment of the diagnosis of cryptococcosis; 6.4% (7/110) received less than 7 days of treatment, transferred to other health-care centers, and lost to follow-up; 80.9% (89/110) patients received more than 7 days of antifungal treatment and were discharged home with maintenance treatment. In all 89 cases administrated with antifungal agents, 19 (21.3%) have received ventriculoperitoneal shunts. All these 89 patients were followed up to 1-year after hospitalization, and 24.7% (22/89) died. Table 5 shows the comparison of the detailed clinical features between the survivors (67 cases) and non-survivors (36 cases).

**TABLE 5:** Characteristics of 103 survivors and non-survivors with cryptococcal meningitis.

	Total	Survival	Dead	*p*
**No. of cryptococcal meningitis cases**	103^*a*^	67	36	
*Cryptococcus* species (%)				0.625
*C. neoformans s.s.*	98 (95.1)	64 (95.5)	34 (94.4)	
*C. deneoformans*	1 (1.0)	1 (1.5)	0	
*C. deuterogattii*	4 (3.9)	2 (3.0)	2 (5.6)	
**Demographic features**				
**Gender**				
Male (%)	60 (58.3)	39 (58.2)	21 (58.3)	1
Female (%)	43 (41.7)	28 (41.8)	15 (41.7)	1
Age [mean (SD)]	47.7 (16.5)	45.5 (17.1)	49.9 (14.7)	0.195
Contact to pigeon droppings = Yes (%)	8 (7.8)	5 (7.5)	3 (8.3)	1
**Underlying status**				
Hepatitis and liver cirrhosis (%)	13 (12.6)	10 (14.9)	3 (8.3)	0.516
Autoimmune disorders (including 7 SLE cases) (%)	12 (11.7)	4 (6.0)	8 (22.2)	0.033^#^
CKD (%)	6 (5.8)	2 (3.0)	4 (11.1)	0.216
HIV/AIDS (%)	4 (3.9)	1 (1.5)	3 (8.3)	0.239
Diabetes (%)	5 (4.9)	4 (6.0)	1 (2.8)	0.812
Long-term use of immunosuppressants (%)	3 (2.9)	3 (4.5)	0	0.5
Malignancy (%)	2 (1.9)	1 (1.55)	1 (2.8)	1
Pregnancy (%)	2 (1.9)	2 (3.0)	0	0.766
Transplant recipient (%)	1 (1.0)	0	1 (2.8)	0.751
No underlying diseases (%)	40 (38.7)	29 (43.3)	11 (30.6)	0.206
**Clinical presentations**				
Altered mental status (%)	9 (8.7)	4 (6.0)	5 (13.9)	0.322
Fever/chill (%)	49 (47.6)	31 (46.3)	18 (50.0)	0.877
Septic shock (%)	1 (1.0)	0	1 (2.8)	0.751
Seizures (%)	1 (1.0)	1 (1.5)	0	1
Headache (%)	80 (77.7)	53 (79.1)	27 (75.0)	0.819
Stiff neck (%)	43 (41.7)	27 (40.3)	16 (44.4)	0.844
Nausea/vomiting (%)	31 (30.1)	19 (28.4)	12 (33.3)	0.764
Visual disturbance (%)	9 (8.7)	7 (10.4)	2 (5.6)	0.637
Speech difficulties (%)	2 (1.9)	1 (1.5)	1 (2.8)	1
Palsies (%)	4 (3.9)	2 (3.0)	2 (5.6)	0.913
Dizziness (%)	8 (7.8)	5 (7.5)	3 (8.3)	1
Hemiplegia (%)	2 (1.9)	0	2 (5.6)	0.23
Unstable walking (%)	4 (3.9)	3 (4.5)	1 (2.8)	1
Klinefelter sign (%)	23 (22.3)	12 (17.9)	11 (30.6)	0.222
Brinell sign (%)	8 (7.8)	4 (6.0)	4 (11.1)	0.587
**Laboratory tests**				
India ink staining (%)	47 (45.6)	27 (40.3)	20 (55.6)	0.202
**Course of the disease before hospitalization** (days, IQR)		30 (20–60)	22 (15–30)	0.179
**Treatment strategies**				
Shunt-containing regimen (%)^*b*^	20 (19.4)	16 (23.9)	4 (11.1)	0.193
**Mortality (hospitalization to death) (%)**				
30 days-mortality	29 (28.2)		29 (80.6)	
90 days-mortality	33 (32.0)		33 (91.7)	
1 year-mortality	36 (35.0)		36 (100)	
**Not received treatment due to death within three days of admission (%)**	14 (13.6)		14 (38.9)	

**^#^p < 0.05. ^*a*^There are seven subjects of loss to follow-up; ^*b*^shunt: extraventricular drainage and/or ventriculoperitoneal shunts; CKD, Chronic kidney disease; SLE, systemic lupus erythematosus; IQR, interquartile range.**

### Comparison of Clinical and Laboratory Data in 103 Cryptococcal Meningitis Cases Caused by *C. neoformans* Species Complex and *C. gattii* Species Complex, and by ST5 and Non-ST5 *C. neoformans* Species Complex

There were seven cryptococcal meningitis cases lost to follow-up. Then, the clinical and laboratory features between 99 cases by *C. neoformans* species complex and four cases by *C. gattii* species complex were compared, the results were shown in Supplementary Table S1. All four patients infected by *C. gattii* species complex had no underlying diseases, while 36.4% (36/99) patients by *C. neoformans* species complex showed underlying conditions, which is significantly different (*p* < 0.05).

Furthermore, ST5 accounted for 91.9% (91/99) of the 99 *C. neoformans* strains causing meningitis. The differences between ST5 and non-ST5 groups were compared, as detailed in Supplementary Table S2. There were two HIV/AIDS cases in the non-ST5 *C. neoformans* cases (25%), significantly more than those in the ST5 group (2.2%, 2/91) (*p* < 0.05).

### Relationship Between Antifungal Susceptibility Results and Prognosis

As shown in Table 6, for fluconazole-containing regimen, the mortality rate in those infected with non-WT *Cryptococcus* for fluconazole was significantly higher than those with WT *Cryptococcus* (*p* = 0.034, <0.05); however, compared with those in the survival group, the rate of non-WT to 5-flucytosine and non-WT to amphotericin B increased, but statically insignificant. Furthermore, no patient was treated in the present study with voriconazole, itraconazole, and posaconazole.

**TABLE 6:** Relationship between antifungal susceptibility results and prognosis in 110 cryptococcal meningitis subjects.

Treatment regimen	Total	Survival (No. %)	Dead (No. %)	*p-*value
**Amphotericin B containing regimen**	84	64 (76.2)	20 (23.8)	0.588
WT	79	61 (77.2)	18 (22.8)	
Non-WT	5	3 (60.0)	2 (40.0)	
**5-Flucytosine containing regimen**	52	41 (78.8)	11 (21.2)	0.193
WT	48	39 (81.2)	9 (18.8)	
Non-WT	4	2 (50)	2 (50)	
**Fluconazole containing regimen**	57	44 (77.2)	13 (22.8)	0.034^#^
WT	53	43 (81.1)	10 (18.9)	
Non-WT	4	1 (25.0)	3 (75.0)	
**Amphotericin B, fluconazole combined with 5-flucytosine**	29	25 (86.2)	4 (13.8)	0.553
WT	24	21 (87.5)	3 (22.5)	
Non-WT to any of amphotericin B, fluconazole, and 5-flucytosine	5	4 (80.0)	1 (20.0)	
**Amphotericin B combined with fluconazole**	23	16 (69.6)	7 (30.4)	0.067
WT	19	15 (78.9)	4 (21.1)	
Non-WT to amphotericin B or/and fluconazole	4	1 (25.0)	3 (75.0)	
**Amphotericin B combined with 5-flucytosine**	21	16 (76.2)	5 (23.8)	0.429
WT	19	15 (78.9)	4 (21.1)	
Non-WT to amphotericin B or/and 5-flucytosine	2	1 (50.0)	1 (50.0)	
**Fluconazole combined with 5-flucytosine**	2	0	2 (100)	/
WT	1	0	1 (100)	
Non-WT to fluconazole or/and 5-flucytosine	1	0	1 (100)	

**WT, wild-type; Non-WT, non-wild-type. ^#^p < 0.05.**

## Discussion

*Cryptococcus*, usually acquired by inhalation, causes pneumonia and cryptococcemia, and exhibits a propensity to disseminate to the brain and presents as meningitis (Fan et al., 2016; O’Halloran et al., 2017; Cao et al., 2019; Tsai et al., 2019). Cryptococcal meningitis is the most severe and common form of cryptococcosis (Yuchong et al., 2012; Rajasingham et al., 2017). Of 204 cases of cryptococcosis in a US hospital from 1996 to 2009, 62% (126/204) were cryptococcal meningitis (Bratton et al., 2013). In the current retrospective study, we analyzed the clinical features of patients with culture-confirmed meningitis. Meanwhile, we also provided information about the microbial characteristics of *C. neoformans/gattii* species complex isolated.

In this study, of 110 *Cryptococcus* strains from meningitis subjects, *C. neoformans s.s.* predominated (95.5%, 105/110), while *C. deuterogattii* (4, 3.6%) and *C. deneoformans* (1, 0.9%) were rarely identified. By comparison, Chen et al. also showed that 93.0% (120/129) *C. neoformans s.s.* and 7.0% (9/129) *C. gattii* (VGI) were isolated from 1980 through 2006 from cryptococcosis patients in 16 provinces of China (Chen et al., 2008). In Taiwan during 1997–2010, *C. neoformans* and *C. gattii* species complexes accounted for 95.9% (210/219) and 4.1% (9/219), respectively, and the predominant molecular type was also VNI (94.1%, 206/219) (Tseng et al., 2013). However, the species and molecular types distribution of *C. neoformans* and *C. gattii* species complex might be geographically varied, presenting a peculiar epidemiological profile (Chen et al., 2014; Smith et al., 2015; Alves Soares et al., 2019). For example, in 62 *Cryptococcus* strains collected from meningitis cases from 2006 to 2010 in Brazil, *C. gattii* species complex accounted for 21%, tremendously higher than that in mainland China (Matos et al., 2012). In addition, there are several studies in Brazil also showing a high prevalence of *C. neoformans* species complex (Andrade-Silva et al., 2018; Ponzio et al., 2019). Infection due to *C. gattii* species complex is more prevalent in the Northeast of Brazil (Matos et al., 2012), despite has been described in the Southeast (Vilas-Boas et al., 2020). Furthermore, in the current study, all four *C. gattii* species complex strains belonged to VGII molecular type; however, in Colombia, VGII molecular type was identified only in 54.3% *C. gattii* species complex strains (Escandon et al., 2018). Also, *Cryptococcus deuterogattii* molecular type is the most prevalent molecular type in Brazil and it seems to be transmitted from South America to North America (Souto et al., 2016; David and Casadevall, 2019).

ITS and MLST are often applied in the evaluation of the genetic relationship among *Cryptococcus* strains (Fan et al., 2016). Differences in ST distribution have previously been noted across varied populations (Beale et al., 2015; Desnos-Ollivier et al., 2015; Fan et al., 2016). In the current study, *Cryptococcus* strains showed a low degree of genetic diversity, and only seven STs were detected in 110 *Cryptococcus* strains, in which 89.1% were represented by ST5. The ST5 lineage is the predominant ST reported from China. For example, in a study in China, in 303 *C. neoformans* strains from 10 hospitals over 5 years, only 12 STs were identified, and ST5 accounted for 89.2% (272/305) (Fan et al., 2016). In another Chinese study, MLST analysis assigned 41 *C. neoformans* strains into 5 STs, and ST5 accounted for 82.9% (34/41) (Wu et al., 2015). There were two HIV/AIDS patients among the non-ST5 *C. neoformans* cases (25%), significantly more than in the ST5 group (2.2%, 2/91). In addition, in 183 clinical and environmental isolates of *Cryptococcus* strains from Thailand, Southeast Asia, population genetic analyses showed that Thailand isolates from 11 provinces were highly homogenous, consisting of the same genetic background (globally known as VNI) and exhibiting only 10 nearly identical sequence types (STs), with three (STs 44, 45 and 46) dominating their strains (Simwami et al., 2011). In an article in Laos, the strains were dominated (83%) by STs 4 and 6, while in Vietnam, the strains were dominated by the ST4/ST6 (35%) and ST5 (48%) lineages (Thanh et al., 2018). Taken together, the molecular type VNI *C. neoformans* species complex strains with low diversity of STs predominate in China and around Asia, and the predominant STs of *Cryptococcus* strains might differ geographically. The molecular diversity across Southeast Asia has been explained by ecological rather than human host factors (Thanh et al., 2018).

A sex bias is observed in cryptococcal studies. The prevalence of cryptococcosis is consistently common in males in both HIV-positive and -negative patients (Guess et al., 2018). In our study, male patients accounted for 60.9% (67/110). Similarly, male predominance was also shown in Wuhan, China (75.6%, 68/90 cryptococcal meningitis cases) (Cao et al., 2019), in Beijing, China (73%, 38/52 pediatric patients with disseminated cryptococcosis) (Gao et al., 2017), in Colombia (79.6% in the general population and 84.4% in HIV/AIDS patients) (Escandon et al., 2018), and in Brazil (69% in 5,755 recorded deaths related to cryptococcosis) (Alves Soares et al., 2019). Furthermore, in our study, 16.4% (18/110) were elderly patients (≧65 years, 13 males and 5 females). In a study in Taiwan, elderly patients were more vulnerable to cryptococcal meningitis than those aged <65 years, and fewer males were affected in the elderly group (57.9%, 22/38) than in non-elderly group (78.7%, 48/61) (Tsai et al., 2019). The sex bias has been explained by the increased incidence of the HIV epidemic in males (Liu et al., 2017; Guess et al., 2018). Nevertheless, in our study, only 4.55% (5/110) were HIV-positive. Furthermore, *Cryptococcus* species are commonly distributed in the environment and associated with bird excreta (mostly pigeon droppings), soil and wood debris (Alves Soares et al., 2019), and males are more likely to work outside. This could also partially explain the sex disparity (Guess et al., 2018). In the present study, only 9 (8.2%) had been in contact with pigeon droppings, and similarly, only 10 (10/52, 19.2%) cases had a history of exposure to pigeon droppings in pediatric patients in China (Gao et al., 2017). Therefore, the reasonable explanation of male predominance remained elusive.

Cryptococcosis presents in both immunocompromised and immunocompetent subjects (Lahiri et al., 2019). Some cryptococcal meningitis patients might have predisposing factors, which change over time and are geographically varied (O’Halloran et al., 2017; Ellis et al., 2019). The common risk factors for cryptococcosis generally involve HIV/AIDS, organ transplant, corticosteroid use, and malignancy (Hong et al., 2017; Kashef Hamadani et al., 2018; Ellis et al., 2019). In our study, 60% (66/110) of the strains were isolated from patients with apparent risk factors, and the underlying status included hepatitis and cirrhosis (13, 11.8%), autoimmune disorders (12, 10.9%) and diabetes mellitus (5, 4.5%). Comparatively, in Taiwan during 1997–2010, HIV infection was the most common underlying condition (54/219, 24.6%), and among HIV-negative patients, liver diseases (HBV carrier or cirrhosis) were common (30.2%) and 15.4% did not have any underlying condition (Tseng et al., 2013). The current study was conducted in XHCSU, China. It is not a reference service for HIV. HIV-affected patients will be transferred to the specific infectious disease hospital. In another study in China, 71% (91/129) of cryptococcosis cases during 1985–2006 had no apparent risk factor and only 8.5% (11/129) were HIV/AIDS patients (Chen et al., 2008). In addition, in a study in the Southwest of China, among 85 patients with CM were identified, 32 (37.6%) were HIV-uninfected patients (Liu et al., 2017). The less HIV-infected patients might be explained by the bias in the population studied. As previously reviewed, cryptococcosis in non–HIV-infected patients, compared to those HIV-infected, the substantial differences in terms of natural history, clinical course, diagnosis, and outcome should be noted in China, especially the transplant recipients with cryptococcosis (Pappas, 2013).

Different from *C. neoformans* species complex, *C. gattii* species complex-caused cryptococcosis occurs mainly in non-elderly and immunocompetent hosts (Chen et al., 2008; Alves Soares et al., 2019). We compared the clinical and laboratory data of meningitis cases caused by *C. neoformans* and *C. deuterogattii*, and found that four *C. deuterogattii*-infected patients had no underlying diseases, significantly fewer than among *C. neoformans* cases (100 vs. 36.4%). Similarly, in Colombia between 1997 and 2011, 91.1% (41/45) *C. gattii* species complex-caused cases had no predisposing factors and only 6.7% (3/45) were HIV-positive (Lizarazo et al., 2014). It is documented in a previous study that dual tubercular/cryptococcal meningitis was the most frequent (54.0%) and most easily misdiagnosed (95.2%, 40/42) co-infection (Fang et al., 2017). However, in our study, only three tuberculosis patients (2.73%, 3/110) were detected, which could be explained by geographical differences (Fang et al., 2017).

Cryptococcal meningitis is highly lethal without early diagnosis and proper treatment. Its clinical presentation is often not specific (Liu et al., 2017; Alves Soares et al., 2019). In the current study, headache was the most common presentation (85, 77.3%), followed by fever (52, 47.3%), and nausea/vomiting (32, 29.09%), in accordance with another study involving 90 cryptococcal meningitis patients (Cao et al., 2019). In 45 cryptococcosis cases caused by *C. gattii* species complex, their clinical features also included headache (80.5%) and nausea/vomiting (56.1%) (Lizarazo et al., 2014). Additionally, in our study, in 103 patients, the mortality rate was 35.0% (36/103), lower than that in Colombia (47.5%) (Escandon et al., 2018). In Taiwan, patients infected with *C. gattii* species complex, compared to those with *C. neoformans* species complex, were more likely to have a higher 10-week mortality rate (44.4 vs. 22.2%) (Tseng et al., 2013). As is the case with our study, in four *C. deuterogattii* meningitis subjects, two died.

The emergence of *Cryptococcus* strains with resistance or elevated MIC above ECVs is of concern as well. Antifungal susceptibility revealed species-specific differential susceptibility, but the acquired resistance was still an infrequent phenomenon (Hagen et al., 2016). Fluconazole and amphotericin B are the most commonly used antifungal agents for the treatment of cryptococcal meningitis. As shown in Table 4 and Figure 4, 8.6% (9/105), 6.6% (7/105), and 8.6% (9/105) of *C. neoformans s.s.* strains had higher MIC values than the recommended ECVs of fluconazole, 5-fluorocytosine, and amphotericin B, respectively. All four *C. deuterogattii* strains were uniformly designated as WT to the above antifungal agents. In a study from Denmark, all 108 clinical *C. neoformans* and *C. gattii* species complex strains were amphotericin B susceptible (Hagen et al., 2016). In a Chinese study from 10 hospitals over 5 years, among 303 *C. neoformans* species complex strains, 7.6% (23/303) were non-WT to fluconazole, however, seven *C. gattii* species complex strains had WT MICs to all drugs tested except for one *C. gattii* species complex strain with a fluconazole MIC of 16 μg/ml (Fan et al., 2016). By comparison, the susceptibilities of the 52 *Cryptococcus* spp. strains in Zhejiang, mainland China, only one *C. neoformans s.s.* (1/51, 2.0%) was non-WT to amphotericin B (1.0 mg/L) and one (2.0%) non-WT to fluconazole (16 μg/ml) (Fu et al., 2019). Taken together, the elevated MIC values for antifungal agents are rarely observed and partially related to the use of unstandardized regimens, which should attract more attention.

It is recommended that amphotericin B and 5-flucytosine are the preferred agents for the initial or induction therapy, whereas the azoles (especially fluconazole) are generally used in the consolidation and maintenance phases of therapy (Beale et al., 2015; Fan et al., 2016). In 204 adults with cryptococcosis from 1996 to 2009 in the United States of America, 5-flucytosine exposure was demonstrated to be associated with a lower mortality rate (Bratton et al., 2013). In a previous study, the proportion of patients receiving amphotericin B-containing regimen was 70.6% (48/68), and had a lower 30-day mortality rate than those treated with other regimens, but the difference was not statistically significant (16.7%, 8/48 vs. 25.0%, 5/20) (Fu et al., 2019). In our study, the regimen containing amphotericin B, fluconazole and 5-flucytosine demonstrated a lower mortality rate. Furthermore, as revealed in Table 6, the elevated MIC value for fluconazole was statically related to a higher mortality rate (22.8 vs. 18.9%, *P* = 0.034). Nevertheless, the conclusion should be interpreted with caution due to the small size of the present study.

In summary, the present study demonstrated that more *C. neoformans* species complex isolates (mainly *C. neoformans s.s.*) were observed than *C. gattii* species complex (mainly *C. deuterogattii*). Low prevalence of HIV patients with cryptococcal meningitis and relatively high non-WT rates to Amphotericin B and fluconazole in *Cryptococcus* strains in China were also noted. The study will be helpful for understanding the genetic diversity of *Cryptococcus* strains and for decision making in the context of the diagnosis, treatment and prevention strategies in cryptococcosis.

## Data Availability Statement

The raw data supporting the conclusions of this article will be made available by the authors, without undue reservation, to any qualified researcher.

## Ethics Statement

The institutional review boards at the XHCSU approved the study protocol. The written informed consent from participants was waived and the data were analyzed anonymously.

## Author Contributions

YL and MZ isolated the *Cryptococcus* spp. and performed the tests. JY collected the clinical data. ZL collected the laboratory data. YL and BL made substantial contributions to conception and design, and drafted the manuscript. All authors contributed to the article and approved the submitted version.

## Conflict of Interest

The authors declare that the research was conducted in the absence of any commercial or financial relationships that could be construed as a potential conflict of interest.

## Footnotes

1 http://www.cbs.knaw.nl/collections/BioloMICSSequences.aspx

2 http://mlst.mycologylab.org

3 https://itol.embl.de
