# Supplementary figures and images for: Microbiological, Epidemiological, and Clinical Characteristics of Patients With Cryptococcal Meningitis at a Tertiary Hospital in China: A 6-Year Retrospective Analysis
Source: Front Microbiol. 2020 Jul 29;11:1837. doi: 10.3389/fmicb.2020.01837 (PMC7403485; doi:10.3389/fmicb.2020.01837)

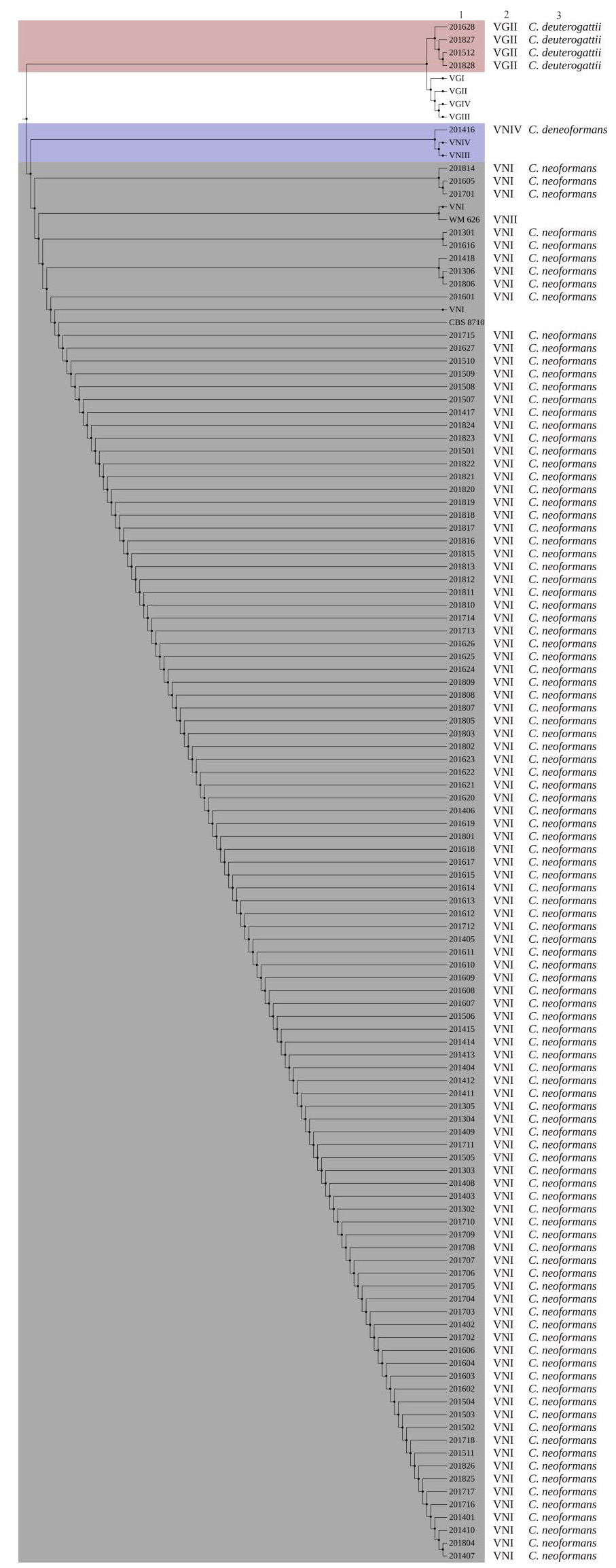

Supplement: Supplementary file 1 [file Image_1.jpeg]
