# Supplementary material for: Microbiological, Epidemiological, and Clinical Characteristics of Patients With Cryptococcal Meningitis at a Tertiary Hospital in China: A 6-Year Retrospective Analysis
Source: Front Microbiol. 2020 Jul 29;11:1837. doi: 10.3389/fmicb.2020.01837 (PMC7403485; doi:10.3389/fmicb.2020.01837)
Supplement: Supplementary file 2 [file Table_1.DOCX]

**Table S1 Characteristics of 103** **meningitis cases caused by *C. neoformans* and *C. gattii* species complex**

|  | **Total** | ***C. neoformans*** **species complex** | ***C. gattii* species complex** | ***p*** |
| --- | --- | --- | --- | --- |
| **No. of cryptococcal meningitis cases** | **103^a^** | **99** | **4** |  |
| **Demographic features** |  |  |  |  |
| Gender |  |  |  |  |
| Male (%) | 60 (58.3) | 57 (57.6) | 3 (75.0) | 0.861 |
| Female (%) | 43 (41.7) | 42 (42.4) | 1 (25.0) | 0.861 |
| Age (mean (SD)) | 47.7(16.5) | 47.8(16.2) | 43.0(22.4) | 0.615 |
| Age distribution(%) |  |  |  | 0.371 |
| ≤14 | 5(14.6) | 5 (5.1) | 0 (0.0) |  |
| 15～24 | 5(14.6) | 4 (4.0) | 1 (25.0) |  |
| 25～34 | 12(11.7) | 11 (11.1) | 1 (25.0) |  |
| 35～44 | 15(14.6) | 15 (15.2) | 0 (0.0) |  |
| 45～54 | 30(29.1) | 30 (30.3) | 0 (0.0) |  |
| 55～64 | 21(20.4) | 20 (20.2) | 1 (25.0) |  |
| ≥65 | 15(14.6) | 14 (14.1) | 1 (25.0) |  |
| Contact to pigeon droppings = Yes (%) | 8 (7.8) | 8 (8.1) | 0 (0.0) | 1.000 |
| **Underlying status** |  |  |  |  |
| Hepatitis and liver cirrhosis (%) | 13 (12.6) | 13 (13.1) | 0 (0.0) | 0.994 |
| Autoimmune disorders (including 7 SLE cases) (%) | 12 (11.7) | 12 (12.1) | 0 (0.0) | 1.000 |
| CKD (%) | 6 (5.8) | 6 (6.1) | 0 (0.0) | 1.000 |
| HIV/AIDS (%) | 4 (3.9) | 4 (4.0) | 0 (0.0) | 1.000 |
| Diabetes (%) | 5 (4.9) | 5 (5.1) | 0 (0.0) | 1.000 |
| Long-term use of immunosuppressants (%) | 3 (2.9) | 3 (3.0) | 0 (0.0) | 1.000 |
| Malignancy (%) | 2 (1.9) | 2 (2.0) | 0 (0.0) | 1.000 |
| Pregnancy (%) | 2 (1.9) | 2 (2.0) | 0 (0.0) | 1.000 |
| Transplant recipient (%) | 1 (1.0) | 1 (1.0) | 0 (0.0) | 1.000 |
| No underlying diseases (%) | 40 (38.7) | 36 (36.4) | 4 (100.0) | 0.042# |
| **Clinical presentations** |  |  |  |  |
| Altered mental status (%) | 9 (8.7) | 8 (8.1) | 1 (25.0) | 0.786 |
| Fever chill (%) | 49 (47.6) | 47 (47.5) | 2 (50.0) | 1.000 |
| Septic shock (%) | 1 (1.0) | 1 (1.0) | 0 (0.0) | 1.000 |
| Seizures (%) | 1 (1.0) | 1 (1.0) | 0 (0.0) | 1.000 |
| Headache (%) | 80 (77.7) | 77 (77.8) | 3 (75.0) | 1.000 |
| Stiff neck (%) | 43 (41.7) | 41 (41.4) | 2 (50.0) | 1.000 |
| Nausea vomiting (%) | 31 (30.1) | 31 (31.3) | 0 (0.0) | 0.434 |
| Visual disturbance (%) | 9 (8.7) | 8 (8.1) | 1 (25.0) | 0.786 |
| Speech difficulties (%) | 2 (1.9) | 2 (2.0) | 0 (0.0) | 1.000 |
| Palsies (%) | 4 (3.9) | 4 (4.0) | 0 (0.0) | 1.000 |
| Dizziness (%) | 8 (7.8) | 7 (7.1) | 1 (25.0) | 0.718 |
| Hemiplegia (%) | 2 (1.9) | 2 (2.0) | 0 (0.0) | 1.000 |
| Unstable walking (%) | 4 (3.9) | 3 (3.0) | 1 (25.0) | 0.363 |
| Klinefelter sign (%) | 23 (22.3) | 22 (22.2) | 1 (25.0) | 1.000 |
| Brinell sign (%) | 8 (7.8) | 7 (7.1) | 1 (25.0) | 0.718 |
| **Laboratory tests** |  |  |  |  |
| India ink staining (%) | 47 (45.6) | 46 (46.5) | 1 (25.0) | 0.739 |
| **Outcome** |  |  |  |  |
| Survival (%) | 67 (65.0) | 65 (65.7) | 2 (50.0) | 0.913 |
| **Mortality (hospitalization to death) (%)** |  |  |  |  |
| 30 d-mortality | 29 (28.2) | 27 (27.3) | 2 (50.0) | 0.672 |
| 90 d-mortality | 33 (32.0) | 31 (31.3) | 2 (50.0) | 0.811 |
| 1 year-mortality | 36 (35.0) | 34 (34.3) | 2 (50.0) | 0.913 |
| **Not received treatment due to death within 3 days of admission (%)** | 14 (13.6) | 12 (12.1) | 2 (50.0) | 0.155 |

**Notes****:** a There are 7 subjects of loss to follow-up; CKD: Chronic kidney disease; SLE: systemic lupus erythematosus.
