# Supplementary material for: Microbiological, Epidemiological, and Clinical Characteristics of Patients With Cryptococcal Meningitis at a Tertiary Hospital in China: A 6-Year Retrospective Analysis
Source: Front Microbiol. 2020 Jul 29;11:1837. doi: 10.3389/fmicb.2020.01837 (PMC7403485; doi:10.3389/fmicb.2020.01837)
Supplement: Supplementary file 3 [file Table_2.DOCX]

**Table S2 Characteristics of 99 meningitis cases caused by ST5 and non-ST5 *C. neoformans* species complex**

|  | **Total** | **ST5** | **Non-ST5** | ***p*** |
| --- | --- | --- | --- | --- |
| **No. of cryptococcal meningitis cases** | **99** | **91** | **8** |  |
| **Demographic features** |  |  |  |  |
| Gender |  |  |  |  |
| Male (%) | 57(57.6) | 53 (58.2) | 4 (50.0) | 0.937 |
| Female (%) | 42(42.4) | 38 (41.8) | 4 (50.0) | 0.937 |
| Age (mean (SD)) | 47.2(16.3) | 47.2 (16.5) | 47.1 (13.3) | 0.986 |
| Age distribution(%) |  |  |  | 0.643 |
| ≤14 | 5(5.1) | 5 (5.5) | 0 (0.0) |  |
| 15～24 | 4(4.0) | 4 (4.4) | 0 (0.0) |  |
| 25～34 | 11(11.1) | 10 (11.0) | 1 (12.5) |  |
| 35～44 | 15(15.2) | 13 (14.3) | 2 (25.0) |  |
| 45～54 | 30(30.3) | 26 (28.6) | 4 (50.0) |  |
| 55～64 | 20(20.2) | 20 (22.0) | 0 (0.0) |  |
| ≥65 | 14(14.1) | 13 (14.3) | 1 (12.5) |  |
| Contact to pigeon droppings = Yes (%) | 8(8.1) | 8 (8.8) | 0 (0.0) | 0.843 |
| **Underlying status** |  |  |  |  |
| Hepatitis and liver cirrhosis (%) | 13(13.1) | 12 (13.2) | 1 (12.5) | 1.000 |
| Autoimmune disorders (including 7 SLE cases) (%) | 12(12.1) | 11 (12.1) | 1 (12.5) | 1.000 |
| CKD (%) | 6(6.1) | 6 (6.6) | 0 (0.0) | 1.000 |
| HIV/AIDS (%) | 4(4.0) | 2 (2.2) | 2 (25.0) | 0.028# |
| Diabetes (%) | 5(5.1) | 5 (5.5) | 0 (0.0) | 1.000 |
| Long-term use of immunosuppressants (%) | 3(3.0) | 3 (3.3) | 0 (0.0) | 1.000 |
| Malignancy (%) | 2(2.0) | 2 (2.2) | 0 (0.0) | 1.000 |
| Pregnancy (%) | 2(2.0) | 2 (2.2) | 0 (0.0) | 1.000 |
| Transplant recipient (%) | 1(1.0) | 0 (0.0) | 1 (12.5) | 0.122 |
| No underlying diseases (%) | 36(36.4) | 33 (36.3) | 3 (37.5) | 1.000 |
| **Clinical presentations** |  |  |  |  |
| Altered mental status (%) | 8(8.1) | 8 (8.8) | 0 (0.0) | 0.843 |
| Fever chill (%) | 47(47.5) | 43 (47.3) | 4 (50.0) | 1.000 |
| Septic shock (%) | 1(1.0) | 1 (1.1) | 0 (0.0) | 1.000 |
| Seizures (%) | 1(1.0) | 1 (1.1) | 0 (0.0) | 1.000 |
| Headache (%) | 77(77.8) | 72 (79.1) | 5 (62.5) | 0.522 |
| Stiff neck (%) | 41(41.4) | 38 (41.8) | 3 (37.5) | 1.000 |
| Nausea vomiting (%) | 31(31.3) | 28 (30.8) | 3 (37.5) | 1.000 |
| Visual disturbance (%) | 8(8.1) | 8 (8.8) | 0 (0.0) | 0.843 |
| Speech difficulties (%) | 2(2.0) | 2 (2.2) | 0 (0.0) | 1.000 |
| Palsies (%) | 4(4.0) | 4 (4.4) | 0 (0.0) | 1.000 |
| Dizziness (%) | 7(2.0) | 6 (6.6) | 1 (12.5) | 1.000 |
| Hemiplegia (%) | 2(2.0) | 2 (2.2) | 0 (0.0) | 1.000 |
| Unstable walking (%) | 3(3.0) | 3 (3.3) | 0 (0.0) | 1.000 |
| Klinefelter sign (%) | 22(22.2) | 20 (22.0) | 2 (25.0) | 1.000 |
| Brinell sign (%) | 7(7.1) | 7 (7.7) | 0 (0.0) | 0.925 |
| **Laboratory tests** |  |  |  |  |
| India ink staining (%) | 46(46.5) | 41 (45.1) | 5 (62.5) | 0.563 |
| **Outcome** |  |  |  |  |
| Survival (%) | 65(65.7) | 62(68.1) | 3(37.5) | 0.174 |
| **Mortality (hospitalization to death) (%)** |  |  |  |  |
| 30 d-mortality | 27(27.3) | 25 (27.5) | 2 (25.0) | 1.000 |
| 90 d-mortality | 31(31.3) | 27 (29.7) | 4 (50.0) | 0.429 |
| 1 year-mortality | 34(34.3) | 29 (31.9) | 5 (62.5) | 0.174 |
| **Not received treatment due to death within 3 days of admission (%)** | 12(12.1) | 10 (11.0) | 2 (25.0) | 0.549 |

**Notes**: CKD: Chronic kidney disease; SLE: systemic lupus erythematosus.
